# ﻿Curating an online checklist for Erica L. (Ericaceae): contributing to and supporting global conservation through the World Flora Online

**DOI:** 10.3897/phytokeys.243.121555

**Published:** 2024-06-21

**Authors:** Alan C. Elliott, Stoffel P. Bester, Ronell R. Klopper, E. Charles Nelson, Michael D. Pirie

**Affiliations:** 1 Royal Botanic Garden Edinburgh, 20a Inverleith Row, Edinburgh, EH3 5LR, Scotland, UK; 2 Foundational Research and Services Directorate, Foundational Biodiversity Sciences Division, South African National Biodiversity Institute, Private Bag x101, Pretoria 0184, South Africa; 3 H.G.W.J. Schweickerdt Herbarium, Department of Plant and Soil Sciences, University of Pretoria, Private Bag X20, Hatfield, Pretoria 0028, South Africa; 4 Tippitiwitchet Cottage, 255A Broadgate, Sutton St Edmund PE12 0LT, Spalding, UK; 5 University Museum, University of Bergen, Postboks 7800, NO-5020 Bergen, Norway; † Deceased

**Keywords:** *Erica*, International register of heather names, World Flora Online

## Abstract

To support the work of the Global Conservation Consortium for *Erica* and update the *Erica* checklist in the World Flora Online (WFO), we have curated the taxonomic backbone in the WFO by expanding it to include updated nomenclatural information from the International Plant Name Index, missing names present in the World Checklist of Vascular Plants (WCVP), the Botanical Database of Southern Africa (BODATSA), and from the “International register of heather names” database, a data source not readily available online. The result is the most robust database of *Erica* names to date, including 851 species, 111 subspecies, 244 varieties, and 2787 synonyms, which is a reliable reference for initiatives such as the *Erica* identification aid, conservation prioritisation, and gap analyses. We disambiguate common orthographic variants within the database and present an overview of these. We also comment on the correct orthography of *E.heleophila* Guthrie & Bolus and *E.michellensis* Dulfer and the validity of *E.tegetiformis* E.G.H.Oliv. are discussed, and the use of *E.adunca* Benth. for a South African species rather than *E.triceps* Link, which is here regarded as insufficiently known and of uncertain application, is clarified.

## ﻿Introduction

*Erica* L., with 851 accepted species (WFO Plant List June 2024), is the second most diverse genus in the Ericaceae after *Rhododendron* L. and is listed amongst the most species-diverse genera of flowering plants (Frodin 2004). The Global Conservation Consortium (GCC) for *Erica* (https://www.globalconservationconsortia.org/gcc/erica/) was established in 2021 (Pirie et al. 2022) to bring together the complementary skills and resources of the world’s *Erica* experts, conservationists, and the botanic garden community for effective conservation to prevent species extinctions. Part of the consortium’s role is to maintain a checklist of accepted species as a baseline to inform and prioritise conservation work. To do this the GCC-*Erica* contributes to the World Flora Online’s (WFO) Ericaceae Taxonomic Expert Network (TEN).

Building and maintaining a checklist of names in a species-rich plant group is rarely trivial, and plants with a history of horticultural innovation present particular challenges. During the nineteenth and the early twentieth centuries, there was often no clear distinction between names given to naturally occurring species and subspecific taxa and the names (often in Latin form) applied in horticulture to plants that would now be regarded as selected cultivars. The problem is exacerbated in *Erica* by the often undocumented and unacknowledged artificial and accidental hybridization of plants in European gardens (Nelson and Pirie 2022). As a result, there are many more validly published names within *Erica* than there are accepted taxa, and the origins of the oldest names can be obscure.

Prior to this work, the most comprehensive checklist of *Erica* names was published in the International Register of Heather Names (IRHN; Nelson and Small 2000; 2004–2005), derived from a highly curated Access 2000® database maintained by The Heather Society and not available online. Many names listed and documented in this work had not been represented in openly accessible databases, and some nomenclatural issues were flagged, but remained unaddressed. During the current work, we resolved several outstanding cases of current use of later homonyms (Nelson et al. 2023). There remains inconsistency in nomenclature between important sources of information, such as conservation threat assessments (Raimondo et al. 2009) and commonly used databases (POWO 2024), and a lack of consensus on synonymy and on numbers of species and subspecific taxa that poses an obstacle to conservation prioritisation, for example in attempts to map taxa from GBIF records or to assign threat status to currently accepted names (Pirie et al. 2024).

The main aim of this work was to create a global checklist for the GCC-*Erica* as part of the World Flora Online. We set out to curate the classification in the WFO backbone, comparing and integrating online resources of BODATSA, IPNI, WCVP, and names data from the IRHN database. We clarify some points of outstanding nomenclatural confusion, including inconsistent use of orthographic variants (Nelson and Oliver 2004) and how these may be impacted by current proposals to amend the botanical code of nomenclature (Mabberly 2020; Van Rijckevorsel 2020).

## ﻿Methods

### ﻿Initial curation

As part of the formation of the WFOTEN for Ericaceae in 2020, the family backbone was supplied by the WFO (WFO Consortium 2019) as a Darwin Core Archive. This seeded a dataset (Elliott et al. 2020), managed in Padme, a taxonomic database system developed by the Royal Botanic Garden Edinburgh. The family data, provided by WFO, was largely based on “The Plant List” (2013) v1.1. For *Erica*, updated nomenclatural records were compared to the “International Plant Names Index” (IPNI). Additional names published since 2012 were added to the backbone. Taxonomic placement of species was checked mainly using literature sources for the “Flora of Southern Africa” region (FSA) (Oliver 1984, 1987, 2000; Oliver and Oliver 2002, 2005; Pirie et al. 2017) and online resources, namely Catalogue of the Plants of Madagascar (Rabarimanarivo et al. 2015) and the Botanical Database of Southern Africa (BODATSA) (South African National Biodiversity Institute 2016). Although the South African data was chiefly accessed through the frontend user interface, the yearly checklist data is available in an archived version (South African National Biodiversity Institute 2024).

In 2022, the WFO’s Rhakhis tool (Hyam et al. 2022; Hyam and Elliott 2023) became available, and data in the WFO taxonomic backbone was synced to the Ericaceae classification from Padme. Curation for Ericaceae then transferred from Padme to Rhakhis.

### ﻿Global conservation consortium for *Erica*

The initial checklist created for the formation of the Global Conservation Consortium for *Erica* (GCC-*Erica*), based on the World Checklist of Vascular Plants (WCVP; Govaerts 2022), was compared to the WFO backbone in Rhakhis. Missing names were added to the WFO backbone and conflicts in classification were highlighted and resolved by referring to the literature or the IRHN.

### ﻿The international register of heather names

In 1970, The Heather Society undertook the role of International Cultivar Registration Authority (ICRA) for the genera *Andromeda* L., *Bruckenthalia* Rchb., *Calluna* Salisb., *Daboecia* D.Don, and *Erica* [these formed a denomination class as defined by the “International code of nomenclature of cultivated plants” 1995 (*ICNCP*) under the International Commission for the Nomenclature of Cultivated Plants]. The published volumes (Nelson and Small 2000, 2004–2005) were derived from a database that included more details of the names, including bibliographic references to descriptions and illustrations, history, and etymology of the individual scientific and horticultural (cultivar) names. To preserve these data, the entire IRHN database has been archived (see below).

The first volume of the “International Register of Heather Names” (IRHN; Nelson and Small 2000) was published in four parts and contained all cultivar and botanical names that had then been traced within the genera *Andromeda*, *Bruckenthalia*, *Calluna*, *Daboecia*, and *Erica* in the broad sense. This list covered species and subspecific taxa of *Erica* species indigenous in Europe, Asia Minor, the Atlantic islands (Azores, Madeira, and the Canary Islands), and Africa north of the Tropic of Cancer, and their natural and man-made hybrids and their cultivars. These are generally known colloquially as “hardy heaths” or “hardy heathers” because the majority can tolerate the relatively cool climate of the northern hemisphere, north of the Tropic of Cancer.

The second volume (Nelson and Small 2004–2005), also published in four parts, contained names for *Erica* species and subspecific taxa indigenous to Africa south of the Tropic of Cancer and the islands of the southern Atlantic and Indian Oceans. Many of these are colloquially known (especially in horticulture) as “Cape heaths” although many do not occur within the Cape provinces of South Africa. This volume also included the names of subspecific taxa, natural and artificial hybrids, and cultivars of the Cape heaths. With the re-circumscription of *Erica* to include previously separate “minor” genera (Oliver 2000), a list of these synonymised genera was included as Annex 1 of Volume 2, and their names, now being synonyms of *Erica* taxa, were also included in that checklist.

Compilation of the IRHN was a major collaborative effort involving members of The Heather Society (1963–2020), particularly its designated registrars, and sister societies in western Europe and North America. Research and publication of the checklist was funded by The Heather Society with additional financial support from the Stanley Smith Horticultural Trust (UK).

Names data from The Heather Society’s Access 2000® database, used to produce the IRHN (Nelson and Small 2000, 2004–2005), were matched against the WFO backbone. Names data were extracted from this database. To aid in name matching, authorships were modified to reflect standard author abbreviations according to Biodiversity Information Standards (TDWG) (formerly the Taxonomic Databases Working Group), and names without an author string were initially excluded. Natural and artificial hybrid names were added to the WFO when missing as these can have nomenclatural significance. Natural hybrids were placed in the classification where appropriate following the IRHN. Artificial hybrids are unplaced and deprecated, as these are outside the scope of the WFO. The status of “deprecated” was introduced primarily as an internal device in the WFO. It is meant in the modern sense of the word, particularly regarding software: “to withdraw official support for or discourage the use of”. Cultivar names were not processed as they also are beyond the scope of the WFO. This process of bringing in historic names from IRHN raised homonym issues among Latin binomials that were known but not yet resolved. The required replacement scientific names have been dealt with by Nelson et al. (2023). These new names were registered with IPNI (2023) as part of the pre-publication process and subsequently added to the WFO database.

An IRHN archive (Elliott et al. 2023) has been created in Zenodo (the general-purpose open repository developed under the European OpenAIRE program). This contains the complete, unedited IRHN database, the two volumes (eight parts) as published by The Heather Society (in pdf format), and a csv file containing WFO IDs linked to botanical names in the IRHN.

### ﻿Correctable orthographic variation

Orthographic variation (particularly in the terminations employed in eponyms and toponyms) has been prevalent in the historic literature for *Erica* (Nelson and Oliver 2004; Turner 2016). Existing WFO records were corrected in accordance with the “International Code of Nomenclature for algae, fungi, and plants” (*ICN*, Shenzhen Code; Turland et al. 2018). Some variants that have featured in botanical and horticultural literature and databases (including SANBI’s Red List of South African Plants for threat status; http://redlist.sanbi.org/genus.php?genus=1820) were added and linked to the currently accepted name. We further considered the future consequences of proposals currently under consideration to modify Article 60.8 of the *ICN* with regard to restricting the correction of names (Proposal 023 by Mabberly 2020; and proposal 024 by Van Rijckevorsel 2020).

### ﻿Unplaced names

In the context of the WFO, unplaced names are those not associated with any taxon as an accepted name or synonym. This could be due a taxonomist having not expressed an opinion on its placement, or the name cannot be resolved to anything in the classification. These records can be found by searching the name parts but are not found by browsing the classification’s hierarchy.

### ﻿Cultivars

Modern cultivar names as defined under the *ICNCP* (Brickell et al. 2009), used in the horticultural industry and in gardens lie beyond the scope of the WFO and were therefore omitted from the checklist. Historic Latin form names applied to horticultural selections were removed from the classification and deprecated when there was little doubt that they did not refer to wild species.

Data on cultivar names published before 2001 can be found in the original Access database format and as a csv file in the Zenodo archive (https://zenodo.org/doi/10.5281/zenodo.10255787) (Elliott et al. 2023). The cultivar list will be added to the Catalogue of Life’s Checklist Bank.

For comparison, the number of accepted species, subspecies, varieties, and synonyms were recorded from the “World Checklist of Vascular Plants” (WCVP; v.11.0) (Govaerts 2023), the “Synonymic Checklists of the Vascular Plants of the World” (v.16.4, Sep 2023) (Hassler 2023), and “The Leipzig Catalogue of Vascular Plants” (v.3.01) (Freiberg et al. 2020) using datasets deposited in the Catalogue of Life Checklist Bank (https://www.checklistbank.org/).

## ﻿Results

The *Erica* checklist, as published in the WFO June 2024 data release, available in a human readable form at https://wfoplantlist.org/, has 851 accepted species (the 852 in the December 2023 release wrongly included *E.perlata* G.Sinclair as Accepted instead of as Unplaced following Nelson et al. 2023). Table 1 compares the number of accepted species, subspecies, and varieties, as well as the number of synonyms and unplaced name records against “The Plant List” v1.1 and the two previous “WFO Plant List” releases.

**Table 1.** Accepted species, subspecies, varieties, and synonyms within *Erica* across versions of “The Plant List” and the “WFO Plant List”.

Version	Species	Subspecies	Varieties	Synonyms	Unplaced names
The Plant List v.1.1 (2012)	1 044	37	37	1 948	178
WFO Plant List (December 2022)	1 061	56	44	2 540	677
WFO Plant List (June 2023)	853	104	199	2 619	729
WFO Plant List (June 2024)	851	111	244	2787	1413

Since the beginning of curation of *Erica* names in early 2023 for GCC-*Erica* and WFO, the number of *Erica* and related synonymised names in Rhakhis has increased by 1012. Removal of the names of artificial (horticultural) hybrids and duplicate name records has reduced the number of accepted species by 208. The process has added more than 800 synonyms by resolving the unplaced names from now synonymised genera and the addition of historic names from the IRHN database. Following published taxonomic accounts and incorporating infraspecific names from BODATSA and IRHN has increased the number of accepted subspecific taxa when comparing the December 2022 to June 2024 WFO release. There are now 111 accepted subspecies compared to 56, an increase of 55 and 244 accepted varieties compared to 44, an increase in 200. The number of unplaced names has also increased.

As of June 2024 there are 1413 unplaced name in the WFO backbone. Unplaced names are mostly historic and still need to be placed in the classification or deprecated within the WFO. These names, especially those of horticultural origin, may not be useful, especially to ecologists and conservationists who are the primary end-users of the WFO. By maintaining these records, however, the wider WFO names database allows for them to be accounted for by those using the data for taxonomic or historical research purposes. Most of these names could not be readily placed using the IRHN database and were deprecated. To reduce confusion, deprecated names are maintained in the WFO database but are not made visible in the public checklist.

### ﻿Number comparison to other Global Lists

Table 2 shows the comparison of numbers of taxa represented across the “World Checklist of Vascular Plants” (WCVP; v.11.0), the “Synonymic Checklists of the Vascular Plants of the World” (v.16.4, Sep 2023), and “The Leipzig Catalogue of Vascular Plants” (v.3.01). There is great variation in the number of names across all taxonomic ranks among these lists, especially in terms of infraspecific taxa and synonym names.

**Table 2.** Accepted species, subspecies, varieties, and synonyms within *Erica* across the four major global checklists.

Global list	Species	Subspecies	Varieties	Synonyms
GCC-Erica Checklist in WFO Plant List (June 2024)	851	111	244	2787
Leizpig Catalogue of Vascular Plants v.3.01 (November 2020)	893	79	116	1196
Synonymic Checklists of the Vascular Plants of the World v.16.4 (September 2023)	839	112	143	2782
World Checklist of Vascular Plants v.11 (20 April 2023)	859	97	247	2688

### ﻿Orthographic variants

Table 3 lists species and hybrid names in *Erica* with orthographic variants that have appeared in botanical and horticultural literature and databases, as well as their currently accepted orthography. An indication is also given for names where the acceptance at the Madrid Nomenclature Section of the International Botanical Congress (IBC; July 2024) of current proposals to amend Article 60.8 of the *ICN*, would result in a reversion to the original spelling as published in the protologue of each name.

**Table 3.** Orthographic variants of species and hybrid names in *Erica* that have appeared in botanical and horticultural literature and databases [as discussed in Nelson and Small (2004–2005) and in Nelson and Oliver (2004) where numerous other orthographic variations are also listed] that were added to WFO and linked to the currently accepted name and spelling [^#^If Proposals 023 (Mabberly 2020) and 024 (Van Rijckevorsel 2020) to emend the *ICN* is ratified at the IBC 2024 the spelling will revert to that given in the ‘Original orthography’ column.].

Correct orthography	WFO ID	Original orthography#	WFO ID	Other orthographic variants	WFO ID
*Ericaaitonii* Masson ex Andrews, non Willd.	wfo-1000061246	*Ericaaitonia*	wfo-0000671323	*Ericaaitoniana*	wfo-0000671324
*Ericaargyraea* Guthrie & Bolus	wfo-0000671411	*Ericaargyrea*	wfo-1000055018		
*Ericabanksii* Andrews, non Willd.	wfo-0000671465	*Ericabanksia*	wfo-1000057523		
Ericabanksiisubsp.comptonii (T.M.Salter) E.G.H.Oliv. & I.M.Oliv.	wfo-0000671466	Ericabanksiasubsp.comptonii	wfo-0001440938		
*Ericabaueri* Andrews	wfo-0000671485	*Ericabauera*	wfo-1200068674		
*Ericabeaumontiae* Andrews	wfo-1000057504	*Ericabeaumontia*	wfo-0000671488	*Ericabeaumontiana*	wfo-0000671489
*Ericablandfordii* Andrews	wfo-0000671517	*Ericablandfordia*	wfo-1000054989		
*Ericabonplandiana* Sims	wfo-0000671531	*Ericabonplandii*	wfo-0000671530		
*Ericabowieana* G.Lodd.	wfo-0000671543	*Ericabowia*	wfo-0000671542		
*Ericacoventryi* Andrews	wfo-0000671767			*Ericacoventrya*	wfo-1000054990
*Ericaetheliae* L.Bolus	wfo-0000671983	*Ericaethelae*	wfo-1000056273	*Ericaethelii*	wfo-1000055005
*Ericaeweriana* Dryand.	wfo-1000057515			*Ericaewerana*	wfo-0000671987
Ericafastigiatavar.coventryi Bolus	wfo-1200011362			Ericafastigiatavar.coventryana	wfo-1200068673
*Ericagordoniae* J.Forbes	wfo-1000057514	*Ericagordonia*	wfo-0000672178	*Ericagordonii*	wfo-1000055024
*Ericaheleophila* Guthrie & Bolus	wfo-0000672224			*Ericaheliophila*	wfo-1000056274
*Ericahendricksei* H.A.Baker	wfo-0000672226			*Ericahendricksi*	wfo-1000056275
*Ericahibbertii* Andrews	wfo-0000672237			*Ericahibbertia*	wfo-1000054992
*Ericairbyana* Andrews	wfo-0000672344			*Ericairbyana*	wfo-1000057513
*Ericalawsonii* Sims	wfo-0000672436	*Ericalawsonia*	wfo-1000056276	*Ericalawsoniana*	wfo-1000056277
*Ericaleei* Andrews	wfo-1000054993	*Ericaleea*	wfo-1000057512		
*Ericalinnaei* Andrews	wfo-0000672483	*Ericalinnaea*	wfo-1000057511		
*Ericamassonii* L.f.	wfo-0000672581	*Ericamassonia*	wfo-0000672580		
*Ericamaximilianii* Guthrie & Bolus	wfo-0000672585			*Ericamaximiliani*	wfo-1000057510
*Ericamichellensis* Dulfer	wfo-1000056285			*Ericamitchellensis*	wfo-1000056285
				*Ericamitchelliensis*	wfo-0000672628
*Ericamonsoniana* L.f.	wfo-0000672640	*Ericamonsoniae*	wfo-1000055014		
*Ericanewdigateae* Dulfer	wfo-0000672700			*Ericanewdigatei*	wfo-1000057505
*Ericanivenii* Andrews	wfo-1000055003	*Ericanivenia*	wfo-0000672714	*Ericanivenia*	wfo-0000672714
*Ericapatersonii* Andrews	wfo-0000672833	*Ericapatersonia*	wfo-1000056281	*Ericapatersonia*	wfo-1000056281
*Ericapetiveri* L.	wfo-0000672890	*Ericapetiveriana*	wfo-0000672895	*Ericapetiveriana*	wfo-0000672895
*Ericaplukenetii* L.	wfo-0000672951			*Ericaplukenetiana*	wfo-0000672950
				*Ericaplukenetia*	wfo-1000057848
Ericaplukenetiisubsp.penicillata (Andrews) E.G.H.Oliv. & I.M.Oliv.	wfo-0000672958			Ericaplukenetiisubsp.penicillata	wfo-0001441063
*Ericapriorii* Guthrie & Bolus	wfo-0000672992			*Ericapriori*	wfo-1000057509
*Ericasainsburyana* Andrews	wfo-0000673173			*Ericasainsburya*	wfo-1000057507
*Ericasalisburii* Andrews	wfo-1000054995	*Ericasalisburia*	wfo-0000673176		
*Ericasavileae* Andrews	wfo-0000673184	*Ericasavilea*	wfo-1000057506	*Ericasavilliae*	wfo-0000673187
				*Ericasavileana*	wfo-0000673185
*Ericashannonii* Andrews	wfo-0000673248	*Ericashannonea*	wfo-1000056279		
*Ericasolandri* Andrews	wfo-0000673268	*Ericasolandra*	wfo-1000054997		
*Ericasparrmannii* L.f.	wfo-1000055043			*Ericasparrmanni*	wfo-0000673276
*Ericathunbergii* Montin	wfo-0000673417			*Ericathunbergia*	wfo-1000056283
*Ericauhrii* Andrews	wfo-1000057518	*Ericauhria*	wfo-0000673487		
*Ericawalkeri* Andrews	wfo-0000673622	*Ericawalkeria*	wfo-1000056280		
*Ericawendlandiana* Klotzsch	wfo-0000673627			*Ericawendlandii*	wfo-1000055071
*Ericazeyheri* Bartl.	wfo-0000673654			*Ericazeyheriana*	wfo-0000673655

### ﻿Nomenclatural notes

Nomenclatural issues have been dealt with by Nelson et al. (2023) as part of the systematics, natural history, and conservation of the *Erica* (Ericaceae) collection. The following three species are further clarified:

***Ericategetiformis* E.G.H.Oliv. in Bothalia 20(1): 46. 1990.**

IPNI: urn:lsid:ipni.org:names:941276-1

WFO: wfo-0000673371

Replaced synonym: Ericasenilisvar.australis Dulfer in Ann. Naturhist. Mus. Wien 66: 32. 1963.

IPNI: urn:lsid:ipni.org:names:77251074-1

WFO: wfo-0000673222

Oliver (1990) raised E.senilisvar.australis Dulfer to species level since it is significantly different from *E.senilis* Klotzsch ex Benth. The epithet ‘*australis*’ was not available for this taxon at species level because of the earlier name *E.australis* L. (in Mant. Pl. Altera: 231. 1771) that remains the valid name for one of the European species. Therefore, Oliver (1990) published the new name *E.tegetiformis* E.G.H.Oliv. for this taxon. We consider this name to be validly published. In other lists it is considered to be not validly published due to the omission of the full reference of the replaced synonym. There is an indirect reference made with the combination “E.senilisKlotzsch ex Benth.var.australis Dulfer: 32 (1963)”. There is only one Dulfer reference in the bibliography of the article by Oliver (1990). While the *ICN* recommends refraining from this practice (see Rec. 41A.1; Turland et al. 2018), it is permissible to have the full and direct reference separate from the newly published name or combination. The year of the journal volume for the Dulfer reference is cited as 1964 (instead of 1963), but we consider this to be a correctable error under Art. 41.6 (Turland et al. 2018). For these reasons, we treat *E.tegetiformis* (in Bothalia 20: 46. 1990) as validly published.

***Ericaheleophila* Guthrie & Bolus in Fl. Cap. (Harvey) 4(1.1): 110. 1905.**

IPNI: urn:lsid:ipni.org:names:328833-1

WFO: wfo-0000672224

This orthographic issue was dealt with in Nelson and Small (2004–2005) but is revisited here. In the key to species and the protologue (Guthrie and Bolus 1905: 19, 110), the species epithet was published as ‘*heliophila*’. However, in the Addenda and Corrigenda preceding the Index and the Corrigenda following the Index in the same volume and section of the “Flora Capensis” (Thiselton-Dyer 1909: 1126, 1168), the epithet was amended to ‘*heleophila*’, changing the meaning of the epithet to “of the marsh” rather than “of the sun”. In the Index (Thiselton-Dyer 1909: 1146), both epithets are listed. It is unclear why this amendment has been largely overlooked. The original publication date of part 1 of volume 4 of “Flora Capensis” was May 1905 (Stafleu and Cowan 1979: 76) and the correction was published in February 1909 (part 6 of the volume), albeit in the same volume and section (volume 4 section 1). The correction of the name *E.heleophila* is an orthographic (potentially, typographic; Nelson and Small 2004–2005) error permissible under Art. 60.1 (Turland et al. 2018).

The WFO ID of the original orthographic variant can be found in the Table 3.

***Ericamichellensis* Dulfer in Ann. Naturhist. Mus. Wien 67: 85. 1963.**

IPNI: urn:lsid:ipni.org:names:329124-1

WFO: wfo-1000055012

*Ericasaxatilis* L.Bolus in Ann. Bolus Herb. 3: 177. 1924. nom illeg. hom. non *Ericasaxatilis* Salisb., Prodr. Stirp. Chap. Allerton: 295. 1796.

IPNI: urn:lsid:ipni.org:names:329562-1

WFO: wfo-0000673189

This orthographic issue was also dealt with by Nelson and Small (2004–2005) but is revisited here. The name *E.saxatilis* L.Bolus (in Ann. Bolus Herb. 3: 177. 1923) is an illegitimate later homonym of the earlier name *E.saxatilis* Salisb. (in Prodr. Stirp. Chap. Allerton: 295. 1796) (= *E.carnea* L.). Dulfer (1963) therefore published a new name for this taxon, namely *E.mitchelliensis*, with that original spelling. The type collection of *E.saxatilis* L.Bolus was given as “Cape Province; South-Western Region; Ceres Div., Mitchells Peak, Mitchells Pass, “growing on rocks, rare,” alt. 4500 ft., fl. Dec. 1920, *T.P. Stokoe 66*” (Bolus 1923: 177). The peak and pass commemorate Charles Cornwallis [baptised Collier] Michell (1793–1851) (Richings 2006), Surveyor-General of the Cape of Good Hope and Superintendent of Works in 1848 when the pass was originally opened (Raper et al. 2014). Dulfer (1963) chose ‘mitchelliensis’ as his epithet, using the “Mitchell” spelling as it was found in the *E.saxatilis* protologue (Bolus 1923), and derived from the label on Stokoe’s specimens.

Dulfer (1963) also constructed the name with an additional “i” before the -ensis. This has been considered a correctable error in previously published works. The corrected orthography, *E.mitchellensis*, is used in the South African National Plant Checklist (South African National Biodiversity Institute 2024) and Red List of South African Plants (Turner 2008).

The IRHN (Nelson and Small 2004–2005) further corrected the name to *E.michellensis* due to the incorrect spellings of “Mitchell’s Peak” and “Mitchell’s Pass”, when they should have been Michell’s Peak and Michell’s Pass as on modern maps of the region. The correction in the IRHN has not been widely adopted or used, but there is nothing in the *ICN* to suggest that Nelson’s entry in the IRHN is incorrect. We therefore suggest that *E.michellensis* is the correct orthography to follow for this name. Both orthographic variants are in the WFO as separate entries and synonymised to *Ericamichellensis*.

The WFO IDs for the orthographic variants can be found in the Table 3.

### ﻿Resolving the application of the name *Ericaadunca* Benth. (1839), rather than *Ericatriceps* Link (1821)

***Ericatriceps* Link in Enum. Hort. Berol. Alt. 1: 371. 1821.**

IPNI: urn:lsid:ipni.org:names:329771-1

WFO: wfo-0000673442

*Ericaadunca* Benth. in Prodr. 7: 618. 1839.

IPNI: urn:lsid:ipni.org:names:328152-1

WFO: wfo-0000671312

Although previously treated as separate species (e.g., in Schumann et al. 1992: 193 and 195), Oliver (in Oliver 2012 and in Oliver and Forshaw 2012) treated these two names as synonymous. The name *E.triceps* is the older of the two names and thus has priority (Art. 11.1; Turland et al. 2018). However, a note in the ID aid from Oliver (Oliver and Forshaw 2012) stated that any type in Berlin was destroyed during the Second World War and the description in the protologue (Link 1821: 371) is insufficient to definitely associate it morphologically with *E.adunca* or any other known species. No type was explicitly designated in the protologue, but original material derived from the plant cultivated at the Berlin Botanical Garden in 1808 can be assumed to have been in Herb. B. We have not traced other original material. Dulfer (1964: 116 no. 370) noted that the habitat of the species was the Cape of Good Hope (“Hab. in promont. bon. sp.”), repeating information published by Link (1821: 371 no. 3731: “Hab. in Pr. b. sp.”) in the protologue. However, Dulfer (1964) did not directly cite any extant specimen as a type.

Nomenclatural resolution depends on the relative use of *E.triceps* and *E.adunca* for the species as currently circumscribed. Use of the older name, *E.triceps*, and its application is uncertain due to the lack of original material and the ambiguity of the description in the protologue (Link 1821: 371). Should *E.triceps* and *E.adunca* be regarded as synonymous, unequivocal use of the younger name, *E.adunca*, would require formal rejection of the older *E.triceps* under Art. 56 (Turland et al. 2018). Such action at this stage would be premature as further investigation might reveal information that can clarify the application of *E.triceps*. We believe the best course of action is to regard *E.triceps* as an insufficiently known name that cannot be applied to any extant taxon with certainty, and we do not treat it as a synonym of *E.adunca*. We apply only the name *E.adunca* to the South African taxon that has previously been treated as either *E.adunca* or *E.triceps*, the latter probably a misapplication.

## ﻿Discussion

The WFO’s Rhakhis tool, which is made available for use by Taxonomic Expert Networks to curate and produce classification, has allowed for a hybrid approach to curate the *Erica* checklist. A mix of batch processing of csv files and manual edits to records through the user interface has allowed multiple collaborators to contribute to the process.

While the number of unplaced names has increased with the inclusion of IRHN data these are mostly historic names that may never be adequately placed in the generic classification due to incomplete descriptions, the absence of supporting herbarium specimens or competent scientific illustrations. Many have been treated by previous authors as, for example, “imperfectly known species” or “supposed hybrids” (Guthrie and Bolus 1905: 310–315) or “Ungenügend bekannte Arten [insufficiently known species]” or putative hybrids (Dulfer 1964: 139–148). Some of the unplaced names can be accounted for by this ambiguity created by the historic usage of Latin names for plants of horticultural origin. Gradually all unplaced names will be re-assessed, and either placed where appropriate in the classification, or deprecated from the main checklist.

While we corrected orthography in the WFO checklist to follow previously corrected versions in the literature, and corrected others (Table 3), we are aware that Proposals 023 (Mabberly 2020) and 024 (Van Rijckevorsel 2020), if passed at the 2024 IBC, may impact those changes. If that is the case, then these will revert to the original author’s spelling (as indicated in Table 3).

Through the inclusion of the names data from IRHN, the resolution of homonym issues by Nelson et al. (2023), and the nomenclatural work highlighted in this paper, we believe the current checklists in the WFO’s December 2023 release is the most robust global checklist for *Erica*. The need to maintain and update the checklist is essential if it is to be the baseline for conservation efforts. We feel that the most sustainable way to achieve this is through continued collaborative contributions to the WFO using the Rhakhis tool.

### ﻿Future work

Immediate work is required to reduce the number of unplaced names, by placing names that can be traced to wild plants in the classification and deprecating those of horticultural origin.

As of June 2024, many names have a taxonomic reference, i.e. the citation from where the taxonomic concept or circumscription is derived (see Berendsohn 1995) but more references are needed. These references are currently omitted because the relevant publications lack a doi or stable URL to link with, which is a requirement for references in Rhakhis. Use of a taxonomic concept reference is implemented throughout the EricaceaeTEN and follows the best practice adopted by the CaryophyllalesTEN (Fassou et al. 2022; Korotkova et al. 2021).

The *Erica* checklist will be continually edited, when appropriate, via the WFO Rhakhis tool to contribute to the EricaceaeTEN, the wider WFO project and support various activities of the GCC-*Erica*. The six-monthly releases, apart from providing achievable deadlines for incremental improvements, also allow for a stable citable taxonomy that can be referenced and compared across time through the WFO Plant List API.

The *Erica* classification was extracted from the December 2023 WFO Plant List release to synchronise the classification in the *Erica* identification aid (Oliver et al. 2024). Synchronisation to future WFO Plant List releases will continue.

In working through the developing WFO pipeline with Catalogue of Life (CoL), the *Erica* checklist will be incorporated into the annual CoL Checklist and from there can be utilised by the Global Biodiversity Information Facility (GBIF).

## ﻿Additional information

### Author ORCIDs

Alan C. Elliott https://orcid.org/0000-0002-8677-5688

Stoffel P. Bester https://orcid.org/0000-0001-6961-5052

Ronell R. Klopper https://orcid.org/0000-0002-0948-5038

E. Charles Nelson https://orcid.org/0000-0002-3909-4388

Michael D. Pirie https://orcid.org/0000-0003-0403-4470

### Data availability

All of the data that support the findings of this study are available in the main text.
